# An evaluation of the stability of image‐quality parameters of Varian on‐board imaging (OBI) and EPID imaging systems

**DOI:** 10.1120/jacmp.v16i2.5088

**Published:** 2015-03-08

**Authors:** Dennis N. Stanley, Nikos Papanikolaou, Alonso N. Gutiérrez

**Affiliations:** ^1^ Department of Radiation Oncology The University of Texas Health Science Center, San Antonio San Antonio TX USA

**Keywords:** IGRT, quality assurance, OBI, EPID

## Abstract

Quality assurance (QA) of the image quality for image‐guided localization systems is crucial to ensure accurate visualization and localization of regions of interest within the patient. In this study, the temporal stability of selected image parameters was assessed and evaluated for kV CBCT mode, planar radiographic k V, and MV modes. The motivation of the study was to better characterize the temporal variability in specific image‐quality parameters. The CATPHAN, QckV‐1, and QC‐3 phantoms were used to evaluate the image‐quality parameters of the imaging systems on a Varian Novalis Tx linear accelerator. The planar radiographic images were analyzed in PIPSpro with high‐contrast spatial resolution $\left(f_{30},f_{40},f_{50}\text{lp}/\text{mm}\right)$ being recorded. For OBI kV CBCT, high‐quality head full‐fan acquisition and pelvis half‐fan acquisition modes were evaluated for uniformity, noise, spatial resolution, HU constancy, and geometric distortion. Dose and X‐ray energy for the OBI were recorded using the Unfors RaySafe Xi system with the R/F High Detector for kV planar radiographic and the CT detector for kV CBCT. Dose for the MV EPID was recorded using a PTW975 Semiflex ion chamber, PTW UNIDOS electrometer, and CNMC Plastic Water. For each image‐quality parameter, values were normalized to the mean, and the normalized standard deviations were recorded to evaluate the parameter's temporal variability. For planar radiographic modes, the normalized standard deviations of the spatial resolution $\left(f_{30},f_{40},\&\;f_{50}\right)$ were 0.015, 0.008, 0.004 lp/mm and 0.006, 0.009, 0.018 lp/mm for the kV and MV, respectively. The normalized standard deviation of dose for kV and MV were 0.010 mGy and 0.005 mGy, respectively. The standard deviations for full‐and half‐fan kV CBCT modes were averaged together. The following normalized standard deviations for each kV CBCT parameter were: 0.075 HU (uniformity), 0.071 HU (noise), 0.006 mm (AP‐geometric distortion), 0.005 mm (LAT‐geometric distortion), 0.058 mm (slice thickness), 0.124 (f50), 0.031 (HU constancy – Lung), 0.063 (HU constancy – Water), 0.020 (HU constancy – Bone), 0.006 mGy (Dose – Center), 0.004 mGy (Dose –Periphery). Using control chart analysis, institutional QA tolerances were reported as warning and action thresholds based on 1σ and 2σ thresholds. A study was performed to characterize the stability of image‐quality parameters recommended by AAPM Task Group‐142 for the Varian OBI and EPID imaging systems. Both imaging systems show consistent imaging and dosimetric properties over the evaluated time frame.

PACS number: 87.10.‐e

## I. INTRODUCTION

With the commercial availability of image‐guided radiation therapy (IGRT) systems, IGRT equipment has been rapidly integrated into radiotherapy clinics. In IGRT, a high standard of image quality assurance (QA) is required to ensure better localization and identification of regions of interest, particularly tumor volumes. Compared to nonimage‐guided radiation therapy, IGRT offers an enhanced delivery accuracy of precise volumetric dose distributions through the use of volumetric or planar X‐ray imaging localization techniques.^(1)^ IGRT also enables better intrafraction and interfraction visualization, identification of the target volume^(2)^ and potentially reduced patient specific PTV (planning target volume) margins due to the monitoring of the target volume throughout treatment.^3^, ^4^, ^5^

In order to ensure functionality and consistency of IGRT equipment, a clinically robust QA program that maximizes image quality and minimizes radiation dose is necessary. The American Association of Physicist in Medicine (AAPM) task groups 142^(5)^ and 179^(6)^ have discussed the capabilities and set basic image‐quality QA procedures for both planar radiographic and cone‐beam computed tomography (CBCT)‐based modalities, respectively. In both reports, the task groups defined specific image‐quality characteristics that were important for each modality. Task Group 142 recommends a QA testing program, frequency, and tolerance values for the planar radiographic modalities,^(5)^ while TG‐179 recommends a similar format for all CBCT based imaging modalities.^(6)^ In both reports, a suggested tolerance of “baseline” was recommended for the majority of the image‐quality parameters. Establishment of the baseline and tolerance level, or temporal variability, of individual image‐quality parameters is considered to be institution‐specific, but neither task group report proposed a protocol for initial setup and monitoring of consistency. With this in mind, the aim of this study was to evaluate the stability of the image‐quality parameters of the Varian On Board imager (OBI) and Varian electronic portal imaging device (EPID), following the guidelines of AAPM TG‐142 and TG‐179. Based on the analysis of the consistency and stability over the evaluated time period, institutional QA tolerances for warning and action thresholds for each imaging quality parameter can be established.

## II. MATERIALS AND METHODS

### A. Materials

#### A.1 Varian On‐Board Imager (OBI v. 1.4.3)

**Figure 1:**
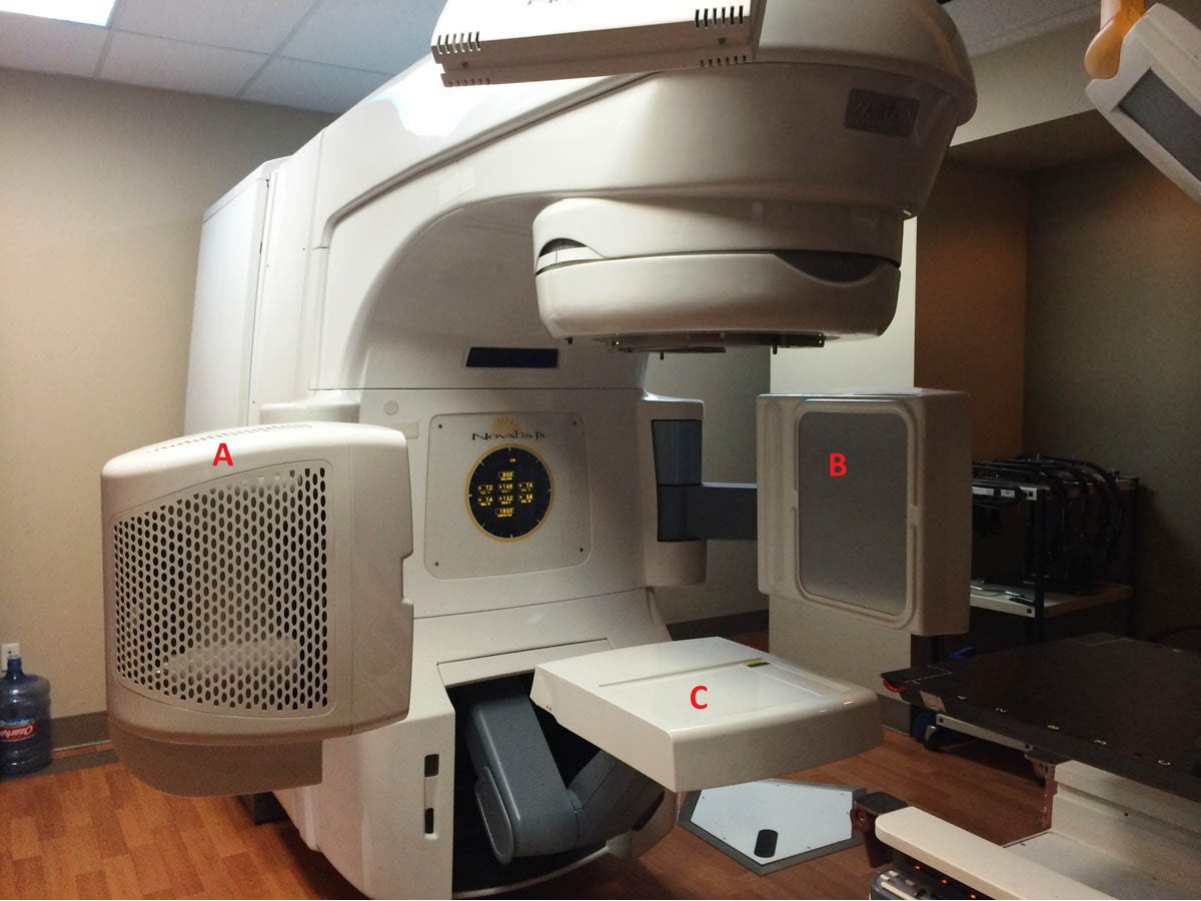
The on board image (OBI) guidance system and EPID image system of the Varian Novalis Tx radiation delivery system are shown: (a) kV X‐ray source of the OBI, (b) a‐Si flat‐panel detector of the OBI, and (c) MV a‐Si EPID mounted to an ExactArm.

The Varian On‐Board Imager (Varian Medical Systems, Palo Alto, CA) consists of two gantry mounted robotic arms called ExactArms that are mounted perpendicularly to the radiation beam, as seen in Fig. 1. Arm A in Fig. 1 is the kilovoltage X‐ray source that has a tube voltage of 40 to 150 kV, while Arm B is an a‐Si flat‐panel detector with an active imaging area of $40\times 30\;{\text{cm}}^{2}$. The OBI has a source to image distance (SID) of 100–182.5 cm depending on the desired scanning characteristics. The OBI consists of three different imaging modalities: a 2D kV planar radiographic (2D kV) mode, a 3D kV cone‐beam computed tomography (CBCT) mode, and a fluoroscopic kV acquisition mode. For the purpose of this study, only the 2D kV planar radiographic and kV CBCT modes will be evaluated. CBCT image sets can be acquired in either half‐fan (pelvis standard) or full‐fan (high quality head) modes with the maximum field of view (FOV) being 45 and 24 cm, respectively.^(7)^ Both kV CBCT acquisition modes have a slice thickness of 1.0–5.0 mm and a reconstructed volume resolution up to 512×512.

#### A.2 Varian aS1000 electronic portal imaging device (EPID)

The Varian aS1000 (PortalVision, Varian Medical Systems, Palo Alto, CA) is an amorphous silicon flat‐panel imaging device mounted on a robotic arm, designated at C in Fig. 1. This arm allows the detector to be positioned at source to EPID distances from 95 cm to 180 cm, with an active imaging area of 40×30 cm.^(8)^ The image matrix is created from an array of 1024×768 photodiodes, giving an effective pixel size of 390 μm at FDD of 150 cm.^(9)^ The EPID has an acquisition rate of 9.574 frames/s, photon energy range of 4–25 MV, and permitted dose rates of 50–600 MU/min.^(10)^ While the EPID can be operated in various acquisition modes, a single exposure, 6 MV planar radiographic mode was used in this study.

#### A.3 The CATPHAN 504 phantom

The CATPHAN 504 (Phantom Laboratory, Salem, NY) was used to evaluate the image‐quality parameters of the kV CBCT for both full‐fan and half‐fan acquisition modes. The CATPHAN is a cylindrical phantom with outer diameter of 20 cm, inner diameter of 15 cm, and four different inserted modules that can evaluate image uniformity, image noise, image hard contrast spatial resolution, HU constancy, geometric distortion, and slice thickness.^(11)^ The CATPHAN was scanned with an image reconstruction of 512×512 pixels, scan width of 16 cm, and FOV of 24 cm or 25 cm, for the full‐fan and half‐fan, respectively. The CATPHAN was chosen for its ease of setup and use, commercial availability (commonly provided with purchase of linear accelerator), and compatibility with PIPSpro software.

#### A.4 QCkV‐1, QC‐3 phantoms and PIPSpro v. 5.0.2

The PIPSpro QA software and phantom package (Standard Imaging, Middleton, WI) was used in this study to analyze the specific image quality parameters for both the OBI and EPID. PIPSpro was chosen because it has a dedicated kV X‐ray phantom (QCkV‐1 Phantom) dedicated MV X‐ray phantom (QC‐3), software tracking capabilities and widespread use for TG‐142 imaging analysis. For the kV and MV planar radiographic modes, the following TG‐142 imaging metrics can be measured and analyzed in PIPSpro using the QCkV‐1 and QC‐3 phantoms: hard contrast spatial resolution, contrast‐to‐noise ratio (CNR), and image noise. For the CBCT, the following TG‐179 imaging parameters can be measured and analyzed in PIPSpro with the CATPHAN phantom: image uniformity, image noise, hard contrast spatial resolution, HU constancy, image geometric distortion, and slice thickness. The QCkV‐1 and QC‐3 phantoms have 11 different regions of interest that contain line pair patterns and materials of varying densities.^(3)^ Having these different regions of the respective phantoms allow the PIPSpro software to evaluate, store, and track the image‐quality parameters over time. The current version (Version 5.0) of PIPSpro software allows the user to either: 1) acquire a food field and an image of the QCkV‐1 or QC‐3 phantoms, or 2) acquire two sequential phantom images for analysis. In this study, the images were evaluated using an acquired food field and one image of the phantom.

#### A.5 Unfors RaySafe Xi R/F and CT detectors

The Unfors RaySafe Xi (Unfors RaySafe AB, Billdal, Sweden) is a comprehensive system of detectors that can perform multiparameter measurements on all X‐ray modalities. The system is composed of a base unit and multiple detectors that are jointly certified by the AALA (American Association for Laboratory Accreditation) and ADCL (American Dosimetry Calibration Laboratory). In this study, the R/F and CT detectors were used in conjunction with the base unit for kV planar radiographic and kV CBCT modes, respectively. The R/F detector is a small, lightweight, portable, and wireless detector capable of simultaneously measuring kVp, dose, dose rate, pulse, pulse rate, dose/frame, time, HVL, total filtration, and waveforms. The CT detector is a 100 mm long hybrid carbon fiber pencil ion chamber capable of measuring dose while actively compensating for both temperature and pressure. The CT detector was used in conjunction with a standard set of acrylic CTDI phantoms with diameters of 32 cm and 16 cm for the half‐fan and full‐fan, respectively. For the purposes of this study, the image parameters evaluated were the dose for kV CBCT, MV, and kV planar radiographic modes and the X‐ray energy for the kV planar radiographic mode.

#### A.6 PTW975 Semiflex ion chamber, PTW UNIDOS^webline^ electrometer, and Plastic Water

The PTW975 Semiflex ion chamber is a waterproof graphite thimble chamber with a vented sensitive volume of 0.3cm^3^ and inner diameter of 5.5 mm. It has a nominal useful energy range from 30 kV to 50 MV photons and 6 MeV to 50 MeV electrons. The PTW UNIDOS^webline^ electrometer is an ADCL‐calibrated reference class electrometer. Plastic Water (CNMC, Nashville, TN) is a water‐equivalent epoxy resin phantom with a density of $1.04\;g/{\text{cm}}^{3}$. A $30\times 30\times 1\;{\text{cm}}^{3}$ slab of Plastic Water was used in conjunction with the PTW975 Semiflex ion chamber and PTW ${\text{UNIDOS}}^{\text{webline}}$ electrometer to measure the imaging dose to the EPID.

### B. Methods

#### B.1 kV planar radiographic

**Figure 2:**
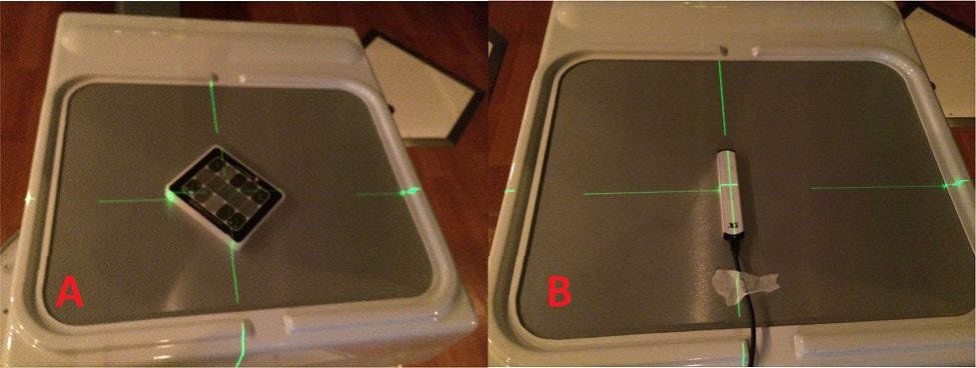
The a‐Si flat‐panel detector of the Varian OBI system is shown with the (a) QCkV‐1 image quality phantom and (b) RaySafe Unfors R/F detector in measurement position.

To evaluate the imaging quality parameters, the QckV‐1 phantom was placed directly onto the face of the OBI detector with the bowtie filters removed and aligned to the room lasers, as seen in Fig. 2(a). One image was acquired with the following settings: 65 kV, 100 mA, and 10 ms.

After removing the QckV‐1 phantom, a second food field image was acquired with the same settings as before, but with an increased field size to irradiate the total active imaging area. The two images were then analyzed in PIPSpro and the high‐contrast spatial resolution was recorded. Each image has three separate values of the high‐contrast spatial resolution ($f_{30},f_{40},f_{50}$ (lp/mm)), which represent the frequencies at 30%, 40%, and 50% maximum of the relative modulation transfer function (RMTF). This method was originally proposed by Droege^(12)^,and the specific equations used were further expanded by Rajapakshe and Luchka.^(13)^ Next, the Unfors RaySafe Xi R/F detector was placed onto the OBI detector, as seen in Fig. 2(b). The process was repeated, with the dose and X‐ray energy being manually recorded after each acquisition.

#### B.2 MV planar radiographic

**Figure 3:**
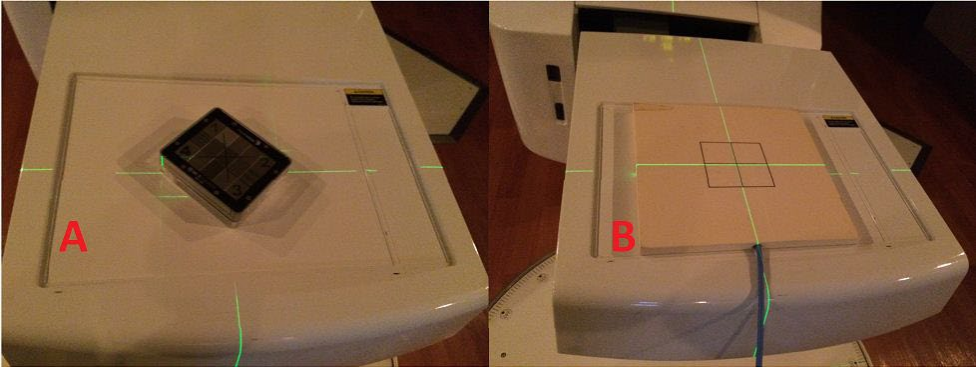
The a‐Si EPID flat‐panel detector is shown with the (a) QC‐3 image‐quality phantom and (b) PTW975 Semiflex Ion Chamber and Plastic Water in measurement position.

To evaluate the imaging quality parameters, the QC‐3 phantom was placed directly onto the face of the EPID and aligned to the room lasers, as seen in Fig. 3(A). The first image was acquired at 6 MV with 4 MU and a $14\times 14\;{\text{cm}}^{2}$ field size. After removing the QC‐3 phantom, a second food field image was acquired with 4 MU and an open field that covered the total active imaging area of the EPID. The two images were then analyzed in PIPSpro and the high‐contrast spatial resolution was analyzed. Next, a 1.0 cm slab of Plastic Water with the PTW975 Semiflex ion chamber was inserted directly into the center was placed onto the face of the EPID, as seen in Fig. 3(b). The chamber was connected to the PTW ${\text{UNIDOS}}^{\text{webline}}$ electrometer and an image was acquired with 4 MU and $10\times 10\;{\text{cm}}^{2}$ field size. The reading was corrected and manually recorded, according to TG‐51^(14)^ protocol, for the dose to the measured point using the following formula:

(1) $$D=M_{RAW}*P_{ion}*P_{TP}*P_{elec}*P_{pol}*k_{Q}*N_{D,wCo60}$$

#### B.3 kV CBCT

**Figure 4:**
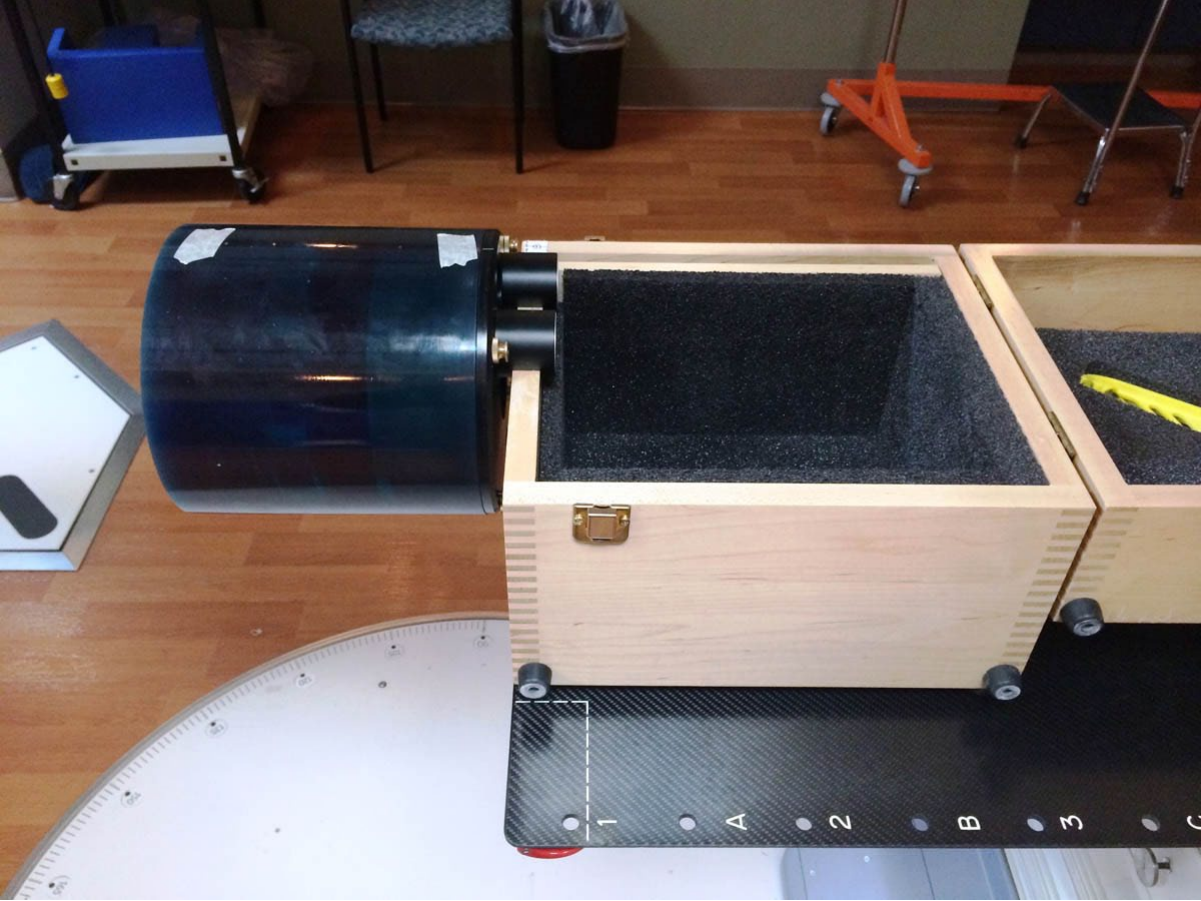
The CATPHAN 504 volumetric image‐quality phantom positioned for evaluation of the half‐fan and full‐fan kV CBCT modes. The CATPHAN is a cylindrical phantom with different inserted modules to evaluate image uniformity, image noise, high‐contrast spatial resolution, HU constancy, image geometric distortion, and image slice thickness.

For the kV CBCT image‐quality parameters, the CATPHAN was cantilevered over the edge of the couch, as seen in Fig. 4. The CATPHAN was leveled and positioned to the imaging isocenter with the aid of the in room localization lasers. One kV CBCT scan per image setting was acquired. Table 1 shows the scanning parameters for each of the acquisition modes.

The image volumes were exported via DICOM protocol and then analyzed in PIPSpro with specific image‐quality parameters being evaluated. Imaging dose was measured using 32 cm and 16 cm diameter standard acrylic CTDI phantoms for the half‐fan and full‐fan modes, respectively.^(15)^ The dose was measured at the center and 12 o'clock periphery position using the Unfors RaySafe Xi CT detector and was manually recorded after each scan. Measurements were specifically acquired at the selected periphery position to ensure full chamber irradiation conditions under both half‐fan and full‐fan modes.

**Table 1:** kV CBCT image scanning parameters using the Varian OBI system. The values for tube voltage, current, and pulse length were customized specifically for our quality assurance scanning protocols

	*Half‐Fan*	*Full‐Fan*
CBCT Mode	pelvis	high quality head
Patient Orientation	head first supine	head first supine
Diameter PA and LR (FOV)	26.0 cm	24.0 cm
Reconstructed Volume	512×512	512×512
Slice Thickness:	2.5 mm	2.5 mm
Width (scan range)	16 cm	16 cm
Tube Voltage (kVp)	85	85
Tube Current (mA)	25	25
Pulse Length (ms)	8	8

For statistical analysis, the QI Macros (KnowWare International, Denver, CO) add‐on statistical analysis package (v.2010.11) was used in Microsoft Excel. The variable control charts modules were used to analyze quality control processes using an X‐bar chart —individual moving range chart test. The software provides control limits for the data and establishes which data points are in control and out‐of‐control processes.

## III. RESULTS

**Figure 5:**
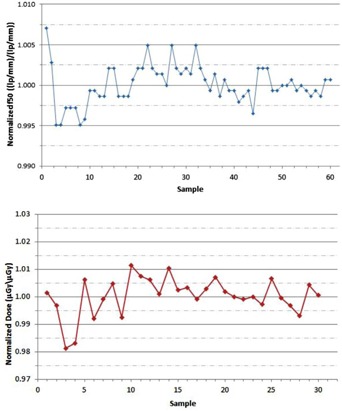
The run chart of the normalized $f_{50}$ values (top) measured with the QCkV‐1 phantom and PIPSpro software is shown for the kV planar radiographic mode of the Varian OBI. The run chart of the normalized image dose values (bottom) measured with the Unfors RaySafe Xi R/F detector is shown for the kV planar radiographic mode of the Varian OBI.

A total of 60 measurements were performed on a single Varian Novalis Tx linear accelerator over a four month period without adjustment or recalibration to the OBI system or the EPID system during the evaluated time frame. For each image‐quality parameter, measured values were normalized to the mean and the standard deviations were recorded. Table 2 shows the standard deviations of all the image‐quality parameters evaluated for the kV planar radiographic, MV planar radiographic, and kV CBCT modes. Run charts were created for each of the evaluated parameters to characterize the temporal variability of each parameter over the evaluated time period and establish upper and lower control limits. Figure 5 shows a sample run chart for the normalized $f_{50}$ and normalized dose values of the planar kV planar radiographic mode. In general, all of the data for the other evaluated parameters showed similar temporal trending.

**Table 2:** Normalized standard deviations of the image‐quality parameters evaluated for the Varian OBI and EPID system. Measurement units denoted in table for each parameter represent the unit used in acquiring raw data. Table results, however, are unit‐less normalized standard deviations

*Planar Radiographic*			*kV CBCT*		
	*kV*	*MV*		*Full‐Fan*	*Half‐Fan*
Spatial resolution (lp/mm)			Spatial resolution (lp/mm) f		
$f_{30}$	0.015	0.006	$f_{30}$	0.087	0.110
$f_{40}$	0.008	0.009	$f_{40}$	0.086	0.116
$f_{50}$	0.004	0.018	$f_{50}$	0.074	0.173
Dosimetric			HU Constancy (HU)		
Dose (μGy)	0.010	0.005^a^	Lung	0.023	0.038
Voltage (kVp)	0.010		Water	0.065	0.058
			Bone	0.020	0.020
			Geometric Distortion (cm)		
			AP	0.005	0.006
			LAT	0.005	0.005
			Mean slice thickness	0.056	0.059
			Dose (mGy)		
			Center	0.004	0.007
			Periphery	0.004	0.003
			Uniformity (HU/HU)	0.061	0.090
			Noise (HU)	0.063	0.079

^a^ Dose mGy.

With the run chart analysis, the variability of each image‐quality parameter can be evaluated to establish tolerance thresholds based on 1σ and 2σ standards. If a warning threshold of 1σ is set, 68% of the readings will fall within the acceptable, no action range. Values outside of the 1σ threshold but within 2σ threshold will fall within the warning range (i.e., 27% of readings fall within the warning range). The warning threshold is chosen to alert the user of a potential abnormal deviation of that image‐quality parameter. A single measurement deviation should not require an action to be taken, but should serve to provide an alert such that the specific parameter be monitored closely. If the image‐quality parameter value exceeds the 2σ threshold, the parameter value is significantly different from the intrinsic variation of the temporal data and should serve as an action threshold since the deviation is true. The action to be taken is dependent upon the underlying cause of the deviation and the clinical impact of the deviation. Table 3 shows the warning and action tolerances adopted in our institution for the kV/MV planar radiographic modes. 4, 5 show the warning and action tolerances adopted in our institution for the full‐fan and half‐fan kV CBCT modes, respectively.

**Table 3:** Warning and Action thresholds for planar radiographic modes based on run chart analysis

	*kV*			*MV*	
	*Warning*	*Action*		*Warning*	*Action*
$f_{30}$ ^a^	2%	4%	$f_{30}$	2%	4%
$f_{40}$ ^a^	1%	3%	$f_{40}$	1%	3%
$f_{50}$ ^a^	1%	3%	$f_{50}$	1%	3%
Dose (μGy)	1%	2%	Dose (μGy)	1%	2%
Energy (kV)	1%	2%			

^a^ $f_{x}$ parameters measured in units of (lp/mm); sample size of 60 measurements taken over four‐month time period.

**Table 4:** Warning and Action thresholds for full‐fan kV CBCT mode based on run chart analysis

	*Warning*	*Action*
Uniformity (HU/HU)	6%	12%
Noise (HU)	6%	12%
Spatial Resolution (lp/mm)		
$f_{30}$	9%	18%
$f_{40}$	9%	18%
$f_{50}$	8%	16%
HU Constancy (HU)		
Lung	3%	6%
Water	6%	12%
Bone	2%	4%
Geometric Distortion (mm)		
AP	1%	2%
LAT	1%	2%
Slice thickness (mm)	6%	12%
Dose (mGy)		
Center	1%	2%
Periphery	1%	2%

Sample size of 60 measurements taken over four‐month time period.

**Table 5:** Warning and Action thresholds for half‐fan kV CBCT mode based on run chart analysis

	*Warning*	*Action*
Uniformity (HU\HU)	9%	17%
Noise (HU)	8%	16%
Spatial Resolution (lp/mm)		
$f_{30}$	10%	20%
$f_{40}$	11%	22%
$f_{50}$	17%	34%
HU Constancy (HU)		
Lung	4%	8%
Water	6%	12%
Bone	2%	4%
Geometric Distortion (mm)		
AP	1%	2%
LAT	1%	2%
Slice thickness (mm)	6%	12%
Dose (mGy)		
Center	1%	2%
Periphery	1%	2%

Sample size of 60 measurements taken over four‐month time period.

## IV. DISCUSSION

With the growing prevalence of IGRT treatments, the ability to determine and monitor the stability of imaging systems is important to ensure robust consistency of the overall imaging quality. This is of utmost significance when imaging is used for adaptive radiotherapy since more restrictive constraints are placed on the image quality over time due to its use in the accuracy of dose calculations and, ultimately, in the clinical judgment of the prescribing physician. AAPM TG‐142 and TG‐179 aid in addressing these concerns by recommending a rigid structure of annual, monthly, and daily QA assessments of specific image‐quality parameters for universal imaging systems. More recently, AAPM has published IGRT Medical Physics Practice Guidelines (MPPGs) which are focused on the commissioning and quality assurance of X‐ray based IGRT radiotherapy systems.^(16)^ In this, a concise, but comprehensive review of key minimal practices for QA of an IGRT system is outlined. Unfortunately, tolerance values for deviations of the various image‐quality parameters under TG‐142 and TG‐179 are not defined and are left to the discretion of the center and qualified medical physicist. Interestingly enough, the MPPGs have now recommended specific tolerance values for the imaging dose based on modality; however, there are still no recommended tolerances for specific image‐quality parameters.

To obtain these tolerances, centers will commonly assess these image‐quality parameters at their recommended temporal frequency, trend their behavior over the course of time, and determine their tolerance based on an internal mechanism. This study was performed to develop an institutional image‐quality assurance protocol with formalized tolerances based on the temporal behavior using commercially available QA devices and software employing the TG‐142 and TG‐179 guidelines. A secondary objective was to report our tolerance values of key image‐quality parameters for the OBI and EPID imaging systems of the Novalis Tx, as this has not been published to date.

While there are a variety of commercial QA packages (hardware and software) available for establishing baselines and trending data, a goal of this study was to characterize the behavior of the imaging systems and provide tolerance values for the evaluated image‐quality parameters such that they can serve as a guide for centers looking to initiate a similar imaging QA program. The understanding, of course, is that with time and more data the individual center will be able to further refine their own tolerances based on the behavior of their specific system. Additionally, it is important to note that the results presented here were obtained with the specific imaging QA hardware and software as previously mentioned in the Materials section. Different centers will inherently possess various evaluation tools for imaging QA analysis; however, if the measurement sensitivities of other evaluation tools are similar, the results shown in this study should apply.

To further generalize this, for each image‐quality parameter, values were normalized to the mean and run chart analyses were conducted with normalized values. In doing so, the dependency of tolerance values on evaluation tools is further minimized. 3, 4, 5 show the warning and action thresholds as determined from the 1σ and 2σ values of the run chart analysis for the planar radiographic, half‐fan, and full‐fan kV CBCT modes. Using these findings, a comprehensive image‐quality QA schedule was formulated based on the TG‐142 schedule. A summarized version of the tolerance data is shown in 6, 7 for the planar radiographic and kV CBCT modes, respectively.

To establish the warning (1σ) and action levels (2σ) of each image‐quality parameter, the methodology used is based on techniques commonly used in production processes.^17^, ^18^ The approach is to produce line charts of the variability in each image‐quality parameter over the evaluated time period. With the assumption that the parameter values are approximately distributed normally, control limits based purely on the behavior of the variability can be generated. Applying the central limit theorem, an upper/lower control limit of 2σ will dictate that 95% of the parameter values will fall within the control limits. In doing so, the system can be characterized as a system that is in control‐reduced variability. Any values outside of the control limits are classified as out‐of‐control and are statistically significant from the mean — at a p‐value of 0.05. Establishing these control limits is crucial in the imaging QA process as they help identify values that are out‐of‐control and need to be investigated.

It is crucial to make clear that the tolerance values and thresholds set forth in this study are strictly based on the observed behavior of each individual image‐quality parameter over the specific evaluated time period. The length of the time period selected sets the warning and action thresholds, and, if a relatively short time period is selected, this may result in too narrow thresholds.

**Table 6:** Institutional imaging QA threshold tolerances for MV/kV planar radiographic modes

*Frequency*	*Quality Metric*	*Quality Check*	*Tolerance Level (Percent of Baseline)*	
			*Warning*	*Action*
Monthly	High contrast spatial resolution (1p/mm)	$f_{30}$	>2%	>4%
		$f_{40}$	>2%	>4%
		$f_{50}$	>2%	>4%
Annual	Imaging quality	Dose Energy	>1%	>2%
		Energy	>1%	>2%

**Table 7:** Institutional imaging QA threshold tolerances for kV CBCT mode

*Frequency*	*Quality Metric*	*Quality Check*	*Tolerance Level (Percent of Baseline)*	
			*Warning*	*Action*
		Geometrical accuracy	>1%	>2%
		Spatial resolution	>10%	>20%
Monthly	Imaging quality	Uniformity	>7%	>14%
		Noise	>7%	>14%
		HU stability	>6%	>12%
Annual	Imaging quality	Dose	>1%	>2%

Using a quality control mindset, the upper and lower control limits are strictly dependent on the observed behavior of the image‐quality parameter rather than on a threshold derived based on a specific clinical impact. More specifically, for example, the $f_{50}$ parameter that monitors the image hard contrast spatial resolution could potentially deviate by more than 2σ in any given month; however, the clinical impact of this deviation may ultimately lead to a minimal impact on pretreatment patient positioning. Unfortunately, there is little literature providing guidance on the clinical impact due to variation in specific image‐quality parameters regarding radiotherapy applications — with the exception of HU number constancy as researchers have shown dosimetric calculation errors do arise due to HU deviations.^(19)^ Nonetheless, if a measurement of an image‐quality parameter falls outside the control limits, the corrective actions to be taken may be simple or complex. Changes in certain image‐quality parameters may simply mandate a recalibration of the imaging hardware, others may mandate replacement of hardware components due to immediate failure, or others may simply be detecting a slow deterioration certain components. Ultimately, the course of action necessary to correct any deviation above 2σ will be situation‐specific and dependent on multiple aspects.

## V. CONCLUSIONS

A study was performed using commercially available imaging QA phantoms and software, as well as diagnostic radiation dosimeters to assess the stability of image‐quality parameters, as recommended by TG‐142 and TG‐179, for the Varian OBI and EPID imaging systems. Both systems show consistent imaging and dosimetric properties over the four‐month evaluation time frame. Using the results of the study, a monthly and annual imaging QA schedule, with suggested tolerance values for the Varian OBI and EPID imaging systems, was established.

## Supporting information

Supplementary Material

## References

[1] Sorcini B and Tilikidis A . Clinical application of image‐guided radiotherapy, IGRT (on the Varian OBI platform). Cancer Radiother. 2006;10(5):252–57. 16884940 10.1016/j.canrad.2006.05.012

[2] Bissonnette JP , Moseley D , White E , Sharpe M , Purdie T , Jaffray DA . Quality assurance for the geometric accuracy of cone‐beam CT guidance in radiation therapy. Int J Radiat Oncol Biol Phys. 2008;71(1 Suppl):S57–S61. 18406939 10.1016/j.ijrobp.2007.06.086

[3] McBain CA , Henry AM , Sykes J , et al. X‐ray volumetric imaging in image‐guided radiotherapy: The new standard in on‐treatment imaging. Int J Radiat Oncol Biol Phys. 2006;64(2):625–34. 16343802 10.1016/j.ijrobp.2005.09.018

[4] Mackie TR , Kapatoes J , Ruchala K , et al. Image guidance for precise conformal radiotherapy. Int J Radiat Oncol Biol Phys. 2003;56(1):89–105. 12694827 10.1016/s0360-3016(03)00090-7

[5] Klein EE , Hanley J , Bayouth J , et al. Task Group 142 report: quality assurance of medical accelerators. Med Phys. 2009;36(9):4197–212. 19810494 10.1118/1.3190392

[6] Bissonnette JP , Balter PA , Dong L , et al. Quality assurance for image‐guided radiation therapy utilizing CT‐based technologies: a report of the AAPM TG‐179. Med Phys. 2012;39(4):1946–63. 22482616 10.1118/1.3690466

[7] Song WY , Kamath S , Ozawa S , et al. A dose comparison study between XVI and OBI CBCT systems. Med Phys. 2008;35(2):480–86. 18383668 10.1118/1.2825619

[8] Menon GV and Sloboda RS . Quality assurance measurements of a‐Si EPID performance. Med Dosim. 2004;29(1):11–17. 15023388 10.1016/j.meddos.2003.09.002

[9] Njeh CF , Caroprease B , Desai P . A simple quality assurance test tool for the visual verification of light and radiation field congruent using electronic portal images device and computed radiography. Radiat Oncol. 2012;7:49. 22452821 10.1186/1748-717X-7-49 PMC3337228

[10] Rajapakshe R , Luchka K , Shalev S . A quality control test tool for electronic portal imaging devices. Med Phys. 1996;23(7):1237–44. 8839419 10.1118/1.597866

[11] Chan MF , Yang J , Song Y , Burman C , Chan P , Li S . Evaluation of imaging performance of major image guidance systems. Biomed Imaging Interv J. 2011;7(2):e11. 22287985 10.2349/biij.7.2.e11 PMC3265149

[12] Droege RT . A practical method to routinely monitor resolution in digital images. Med Phys. 1983;10(3):337–43. 6877181 10.1118/1.595263

[13] Rajapakshe R , Luchka K . Quantitative image analysis for the routine quality control of electronic portal imaging devices In: Faulkner K, Carey B, Crellin A, Harrison RM Quantitative imaging in oncology. Proc. LH Gray Conf. on Quantitative Imaging in Oncology, Newcastle April 1995. London: BIR; 1996 p. 103–05.

[14] Almond PR , Biggs PJ , Coursey BM , et al. AAPM's TG‐51 protocol for clinical reference dosimetry of high‐energy photon and electron beams. Med Phys. 1999;26(9):1847–70. 10505874 10.1118/1.598691

[15] Osei EK , Schaly B , Fleck A , Charland P , Barnett R . Dose assessment from an online kilovoltage imaging system in radiation therapy. J Radiol Prot. 2009;29(1):37–50. 19225181 10.1088/0952-4746/29/1/002

[16] Fontenot JD , Alkhatib H , Garrett JA , et al. AAPM Medical Physics Practice Guideline 2.a: Commissioning and quality assurance of X‐ray‐based image‐guided radiotherapy systems. J Appl Clin Med Phys. 2014:15(1):3–12. 10.1120/jacmp.v15i1.4528 PMC5711227 24423852

[17] Perla RJ , Provost LP , Murray SK . The run chart: a simple analytical tool for learning from variation in healthcare processes. BMJ Qual Saf. 2011;20(1):46–51. 10.1136/bmjqs.2009.037895 21228075

[18] Ott E . Process quality control: troubleshooting and interpretation of data. New York: McGraw‐Hill Book Company; 1975 p. 34–44.

[19] Langen KM , Papanikolaou N , Balog J , et al. QA for helical tomotherapy: report of the AAPM Task Group 148. Med Phys. 2010;37(9):4817–53. 10.1118/1.3462971 20964201

