# Supplementary material for: An evaluation of the stability of image‐quality parameters of Varian on‐board imaging (OBI) and EPID imaging systems
Source: J Appl Clin Med Phys. 2015 Mar 8;16(2):87–98. doi: 10.1120/jacmp.v16i2.5088 (PMC5690094; doi:10.1120/jacmp.v16i2.5088)
Supplement: Supplementary file 1 — Supplementary Material [file ACM2-16-087-s001.docx]

**An Evaluation of the Stability of Image Quality Parameters of Varian On-Board Imaging (OBI) and EPID Imaging Systems**

DN Stanley, MS, N Papanikolaou, Ph.D., AN Gutiérrez, Ph.D.

Department of Radiation Oncology

School of Medicine

Cancer Therapy & Research Center at

The University of Texas Health Science Center San Antonio

San Antonio, TX 78229

USA

Address of Correspondence:

Alonso N. Gutiérrez, Ph.D.

University of Texas Health Science Center San Antonio

School of Medicine

Department of Radiation Oncology (G237)

7979 Wurzbach Rd, MC 7889

San Antonio, TX 78229

U.S.A

Tel: 210.450.1023

Fax: 210.450.1076

E-mail: [gutierrezan@uthscsa.edu](mailto:gutierrezan@uthscsa.edu)

**Abstract**

**Introduction:** Quality assurance (QA) of the image quality for image guided localization systems is crucial to ensure accurate visualization and localization of region of interests within the patient. In this study, the temporal stability of selected image parameters was assessed and evaluated for CBCT mode, planar radiographic kV mode and the radiographic MV EPID mode. The motivation of the study was to better characterize tolerance values for imaging parameters.

**Methods and Materials:** The CATPHAN, QckV-1 and QC-3 phantoms were used to evaluate the image quality parameters. The planar radiographic images were analyzed in PIPSpro™ with high contrast spatial resolution (f30, f40, f50(lp/mm)) being recorded. For OBI CBCT, high quality head Full-Fan acquisition and Pelvis Half-Fan acquisition modes were evaluated for Uniformity, Noise, Spatial Resolution, HU constancy and geometric distortion. Dose and kVp for the OBI were recorded using the Unfors RaySafe Xi system with the R/F High Detector for planar kV and the CT detector for CBCT. Dose for the MV EPID was recorded using a PTW975 Semiflex Ion Chamber, PTW Unidos electrometer and SolidWater™.

**Results:** For each metric, values were normalized to the mean and the standard deviations were recorded. For the planar radiographic spatial resolution the f30, f40, f50 were 0.015, 0.008, 0.004 and 0.006, 0.009, 0.018 for the kV and MV, respectively. The standard deviation of the dose for kV was 0.010 and 0.005 for the MV. The standard deviations for Full and half fan were averaged together and the following standard deviations for each metric were recorded: 0.075(uniformity), 0.071(noise), 0.006(AP-Geometric Distortion), 0.005(LAT-Geometric Distortion), 0.058(mean slice thickness), 0.098(f30),0.101(f40),0.124(f50), 0.031(Lung/PMP-HU constancy), 0.063(Water/poly-HU constancy), 0.020(Bone/Derlin-HU constancy),0.006(Dose-Center), 0.004(Dose-Periphery). Using these, tolerances can be reported as a warning and action threshold of 1σ and 2σ.

**Conclusion:** A study was performed to assess the stability of the basic image quality parameters recommended by TG-142 for the Varian OBI and EPID Imaging systems. The two systems show consistent imaging and dosimetric properties over the evaluated time frame.

**Introduction**

With the commercial availability of Image-guided radiation therapy (IGRT) systems, IGRT equipment has been rapidly integrated into clinics. In IGRT a high standard of image quality assurance (QA) is required to ensure better localization and identification of tumor volumes. Compared to non-image guided radiation therapy IGRT offers an enhanced delivery accuracy of precise volumetric dose distributions through the use of volumetric or planar x-ray imaging localization techniques.^1^IGRT also enables better intra and inter-fraction visualization, identification of the target volume^2^ and reduced patient specific PTV margins due to the monitoring of the target volume throughout treatment.^3,4,5^

In order to ensure functionality and consistency of IGRT equipment a clinically robust QA program that maximizes image quality and minimizes radiation dose should be implemented. The American Association of Physicist in Medicine(AAPM) task groups 142^5^ and 179^6^ have discussed the capabilities and set basic image quality QA procedures for both planar radiographic and CT based modalities respectively. In both reports the task groups defined specific image quality characteristics that were important for each modality. Task group 142 recommended a QA testing program, frequency and tolerance values for the planar radiographic modalities^5^ while TG 179 recommended the same format for all CT based imaging modalities.^6^ In both reports a suggested tolerance of “baseline” was recommended for the majority of the image quality parameters. Establishment of the baseline and specific tolerance levels were considered to be institution-specific but neither purposed a protocol for initial setup and monitoring of consistency. With this in mind, the aim of this study was to evaluate the stability of the imaging components of the Varian On Board imager (OBI) and Varian Electronic portal imaging device (EPID) following the guidelines of AAPM TG 142 and TG 179. Based on the analysis of the consistency and stability over time, we suggested tolerance and action values for the imaging quality metrics based on our institutional results.

**Materials/Methods**

*Materials*

*Varian On-Board Imager (OBI™)*

The Varian On-Board imager (Varian Medical Systems,Palo Alto, CA) consists of two gantry mounted robotic arms called ExactArms that are mounted perpendicularly to the radiation beam as seen in figure 1. Arm A , in figure 1, is the Kilo-Voltage Source that has a tube voltage of 40 to 150kV while Arm B is an a-Si flat panel detector with an active imaging area of 40 x 30 cm. The OBI has a source to image distance (SID) of 100 – 182.5 cm depending on the desired scanning characteristics. The OBI consists of three different imaging modalities: 2D kV planar radiographic (2DkV), 3D Cone-beam Computed Tomography (CBCT) and a fluoroscopic acquisition mode. For the purpose of this study only the 2DkV and CBCT modes will be evaluated. CBCT image sets can be acquired in either Half or Full fan mode with the maximum FOV being 45 and 24 cm respectively^7^. Both CBCT acquisition modes have a slice thickness of 1.0-5.0 mm and a reconstructed volume resolution up to 512x512.

*Varian aS1000 Electronic portal imaging device (EPID)*

The Varian aS1000 (PortalVision, Varian Medical Systems, Palo Alto, CA) is an amorphous silicon flat panel imaging device mounted on a robotic arm designated at C in figure 1. This arm allows it to be positioned at source to EPID distances from 95 cm to 180 cm with an active imaging area of 40 x 30 cm^8^. The image matrix is created from an array of 1024 × 768 photodiodes, giving an effective pixel size of 390 μm at FDD of 150 cm^9^. The EPID has an acquisition rate of 9.574 frames/second, permitted dose range of 4‐25 MV and permitted dose rates of 50-600 MU/min^10^.While the EPID can be operated in many different acquisition modes only MV planar radiographic was used in this study.

*The CATPHAN® 504 Phantom*

The CATPHAN 504 (Phantom Laboratory, Salem, NY) was used to evaluate the image quality parameters of the CBCT for both Full and Half-Fan acquisition modes. The CATPHAN is a cylindrical water equivalent phantom with outer diameter of 20 cm, inner diameter of 15 cm and 4 different inserted modules that can evaluate Uniformity, Noise, Spatial Resolution, HU Constancy, Geometric Distortion and Slick thickness^11^. It was scanned with an image reconstruction of 512x512, scan width of 16 cm, and 24 cm or 25 cm FOV, for the Full-Fan and half-Fan respectively. The CATPHAN was chosen for its ease of setup and use, commercial availability and compatibility with PIPSpro.

*QCkV-1,QC-3 Phantoms and PIPSpro™ V 5.0.2*

The PIPSpro QA software and phantom package (Standard Imaging, Middleton WI) was used in this study to analyze the specific image quality parameters for both the OBI and EPID. PIPSpro was chosen because it has a dedicated kV x-ray phantom (QCkV-1 Phantom), dedicated MV phantom (QC-3), software tracking capabilities and its widespread use for TG-142 imaging analysis. For the 2DkV and MV EPID the following TG-142 imaging metrics can be measured and analyzed PIPSpro with the QCkV-1 and QC-3 phantoms respectively: Spatial resolution, Contrast to noise ratio, and total noise. For the CBCT the following TG-179 imaging metrics can be measured and analyzed in PIPSpro with the CATPHAN: Uniformity, Noise, Spatial Resolution, HU Constancy, Geometric Distortion and Slick thickness. The QCkV-1and QC-3 Phantoms have eleven different regions of interest that contain line pair patterns and materials of varying densities^12^. Having these different regions of the respective phantoms allow the PIPSpro software to evaluate, store and track the image quality parameters over time. The current version (Version 5.0) of PIPSpro software allows the user to either: 1) acquire a flood field and an image of the QCkV-1 or QC-3 phantoms or 2) acquire two sequential phantom images for analysis. In this study, the images were evaluated using an acquired flood field and one image of the phantom.

*Unfors RaySafe Xi R/F and CT Detectors*

The Unfors RaySafe Xi (Unfors RaySafe AB, Billdal, Sweden) is a comprehensive system of detectors that can perform multi-parameter measurements on all x-ray modalities. The system is composed of a base unit and multiple detectors that are ADCL-certified. In this study, the R/F and CT detectors was used in conjunction with the base unit for 2DkV and CBCT respectively. The R/F detector is a small, lightweight, portable, and wireless detector capable of measuring kVp, dose, dose rate, pulse, pulse rate, dose/frame, time, HVL, total filtration and waveforms simultaneously. The CT Detector is a 100mm hybrid carbon fiber ion chamber capable of measuring dose while actively compensating for both temperature and pressure. For the purposes of this study, the parameters evaluated were the dose for both CBCT and 2DkV and kVp the 2DkV only.

*PTW975 Semiflex Ion Chamber, PTW Unidos^webline^ electrometer and SolidWater™*

The PTW975 Semiflex Ion chamber is a waterproof graphite thimble chamber with a vented sensitive volume of 0.3cm^3^ and inner diameter of 5.5mm. It has a nominal useful energy range from 30 kV to 50 MV photons and 6 MeV to 50 MeV electrons. The PTW Unidos^webline^ electrometer is a calibrated reference class dosimeter used for absolute dosimetry. SolidWater™ is a water equivalent epoxy resin phantom with a density of 1.04 g/cm^3^. A 30 x 30 x 1 cm slab of SolidWater was used in conjunction with the PTW975 Semiflex Ion chamber and PTW Unidos^webline^ electrometer to measure the imaging dose to the EPID.

*Methods*

*kV Planar Radiographic*

In order to evaluate the imaging quality parameters the QckV-1 phantom was placed directly onto the face of the OBI detector with the bowtie filters removed and aligned to the room lasers as seen in figure 2A. One image was acquired with the following settings: 65(kV),100(mA),10(ms), 100(SAD). After removing the QckV-1 phantom, a second flood field image was acquired with the same settings as before but an increased field size to irradiate the total active imaging area. The two images were then analyzed in PIPSpro™ and the high contrast spatial resolution (f_30_, f_40_, f_50_(lp/mm)) was recorded. Each Image has three separate values of the high contrast spatial resolution (f_30_,f_40_,f_50_(lp/mm)) which represent the frequencies at 30% , 40% and 50% maximum of the relative modulation transfer function (RMTF).This method was originally purposed by Droege^13^ and the specific equations used were further expanded by Rajapakshe ^14^. Next, the Unfors RaySafe Xi R/F detector was placed onto the OBI detector as seen in Figure 2B. The process was repeated with the dose and kVp being manually recorded after each acquisition.

*MV Planar Radiographic*

In order to evaluate the imaging quality parameters the QC-3 phantom was placed directly onto the face of the EPID and aligned to the room lasers as seen in figure 3A. The first image was acquired with 4 MU and a 14 x 14 cm filed size. After removing the QC-3 phantom, a second flood field image was acquired with 4 MU and an open field that covered the total active imaging area of the EPID. The two images were then analyzed in PIPSpro™ and the high contrast spatial resolution was analyzed the same as with the kV planar radiographic. Next, a 1 cm slab of SolidWater™ with the PTW975 Semiflex Ion Chamber interested directly into the center was placed onto the face of the EPID as seen in Figure3B. The chamber was connected to the PTW Unidos^webline^ electrometer and an image was acquired with 4 MU and 10 x 10 field size. The reading was corrected and manually recorded, according to TG-51^15^ protocol, for the dose to the measured point using the following formula: $D=M_{RAW}*P_{ion}*P_{TP}*P_{elec}*P_{pol}*k_{Q}*N_{D,wCo60}$.

*CBCT*

For the CBCT image quality parameters the CATPHAN was positioned hanging over the edge of the couch as seen in figure 4. The CATPHAN was positioned in the imaging isocenter, with the help of the in room localization lasers, to the third bb in the sagittal position. One scan, per acquisition mode, was acquired and Table 1 shows the scanning parameters for each of the acquisition modes.

The images were then analyzed in PIPSpro with the following metrics being recorded: Uniformity, Noise, High contrast spatial resolution, HU constancy and geometric distortion. Imaging dose was evaluated using a 32 cm and 16cm standard acrylic CTDI phantom for the Half-Fan and Full-Fan respectively^16^. The dose was measured at the center and periphery using the unsfors RaySafe Xi CT Detector and was manually recorded after each scan.

**Results**

For each metric, values were normalized to the mean and the standard deviations were recorded. Table 2 shows the standard deviation for all results.

Using this, tolerances can be reported as a warning threshold of 1σ and an action threshold of 2σ. 1σ warning threshold was chosen to alert the user of potential issues if the parameter is continuously in a warning status for multiple months. The warning threshold does not signify an action threshold requiring immediate attention but rather serves as an alert to monitor that parameter more closely. Table 3 shows the warning and action tolerances for the planar radiographic modalities while tables 4 and 5 show tolerance levels for the Full-Fan and Half-Fan, respectively.

**Discussion**

With the growing prevalence of IGRT treatments, the ability to determine and monitor the stability of imaging systems is important to ensure consistency of the overall imaging quality. This is of utmost significance when the imaging is used for adaptive radiotherapy which places more restrictive constraints on the image quality over time due to its use in dose calculations. AAPM TG-142 and TG-179 alleviates this by recommending a set of annual, monthly and daily QA requirements for different imaging systems. Included in these recommendations are each one of the imaging systems described above. Unfortunately, the tolerance values for the various quality metrics to be examined under TG-142 and TG-179 are not defined and are left to the discretion of the institution and qualified medical physicist. This study was performed to develop an image quality assurance protocol using commercially-available devices and software that allows users to establish an imaging quality assurance program for a modern clinical Linac imaging system which is TG-142 and TG-179 compliant. In addition, it serves to report our institutional tolerance values of key quality metrics of our OBI and EPID systems over the evaluation time period.

While there are a variety of QA packages available for establishing baselines and tolerance values, the goal of the study was to establish a comprehensive imaging quality assurance program for the OBI and EPID imaging systems. That protocol was developed using commercially available QA packages and phantoms that allows users to track the constancy and stability for the Varian OBI and EPID which is both TG-142 and TG-179 compliant. The recorded values from the QA systems were normalized to the mean for each metric and tolerances were determined as the standard deviation of each mean. Table 2 shows the normalized standard deviations for all evaluated metrics of the OBI and EPID. Warning an action levels were set as 1σ and 2σ respectively since any measurement outside of 2σ (95.4%) would demonstrate a significant change to the system.

Using these findings a comprehensive image quality QA schedule was formulated, based on the TG-142 and TG-179, which would allow the user to track the stability of each of the imaging modalities on a modern Linac. Tables 6 and 7 show our Institutional Imaging QA Tolerances for planar radiographic based systems and CBCT systems respectively. It is important to note that the tolerance values set forth in these tables are strictly based on the stability analysis of our systems using the specific methodology and measurement equipment. Although in principle, the tolerance values for other imaging systems should be similar to those in featured tables; our tolerance values may be used as initial values and can be adjusted after the response of a specific system is established.

**Conclusion**

A study was performed using commercially-available imaging QA phantoms and software as well as diagnostic radiation dosimeters to assess the stability of the basic image quality parameters recommended by TG-142 and TG-179 for the Varian OBI and EPID Imaging systems. The two systems show consistent imaging and dosimetric properties over the evaluated time frame. Using the results of the study, an monthly and annual imaging QA schedule and suggested tolerance values for the Varian OBI and EPID Imaging systems, based on the TG-142 and TG-179 guidelines, were established.

**References:**

1. B. Sorcini and A. Tilikidis, “Clinical application of image-guided radiotherapy, IGRT (on the Varian OBI platform),” Cancer Radiother. 10, 252–257 (2006).
2. Bissonnette JP, Moseley D, White E, et al. Quality assurance for the geometric accuracy of cone-beam CT guidance in radiation therapy. Int J Radiat Oncol Biol Phys. 2008;71:S57–S61
3. C. A. McBain, A. M. Henry, J. Sykes, A. Amer, T. Marchant, C. M. Moore, J. Davies, J. Stratford, C. McCarthy, B. Porritt, P. Williams, V. S. Khoo, and P. Price, “X-ray volumetric imaging in image-guided radiotherapy: The new standard in on-treatment imaging,” Int. J. Radiat. Oncol., Biol., Phys. 64, 625–634 (2006).
4. T. R. Mackie, J. Kapatoes, K. Ruchala, W. Lu, C. Wu, G. Olivera, L. Forrest, W. Tome, J. Welsh, R. Jeraj, P. Harari, P. Reckwerdt, B. Paliwal, M. Ritter, H. Keller, J. Fowler, and M. Mehta, “Image guidance for precise conformal radiotherapy,” Int. J. Radiat. Oncol., Biol., Phys. 56, 89–105 (2003).
5. Klein EE, Hanley J, Bayouth J, et al. Task Group 142 report: quality assurance of medical accelerators. Med Phys. 2009;36:4197–4212
6. J.P. Bissonnette, P.A. Balter, L. Dong et al.”Quality assurance for image-guided radiation therapy utilizing CT-based technologies: a report of the AAPM TG-179”Med Phys, 39 (2012), pp. 1946–1963
7. Song et.all, William Y. "A dose comparison study between XVI® and OBI® CBCT systems." Medical Physics (2008): 480-486.
8. Menon GV, Sloboda RS: Quality assurance measurements of a-Si EPID performance. Med Dosim 2004, 29(1):11-7.
9. Njeh Christopher f, Caroprease B,Desai P. “A simple quality assurance test tool for the visual verification of light and radiation field congruent using electronic portal images device and computed radiography” Radiation Oncology 2012, 7:49
10. Rajapakshe, Luchka, et al. 1996. A quality control test tool for electronic portal imaging devices. Medical Physics 23: 1237-44.
11. Chan MF, Yang J, Song Y, Burman C, Chan P, Li S. “Evaluation of imaging performance of major image guidance systems” Biomed Imaging and Intervention J. 2011 Apr-Jun; 7(2): e11.
12. C. A. McBain, A. M. Henry, J. Sykes, A. Amer, T. Marchant, C. M. Moore, J. Davies, J. Stratford, C. McCarthy, B. Porritt, P. Williams, V. S. Khoo, and P. Price, “X-ray volumetric imaging in image-guided radiotherapy: The new standard in on-treatment imaging,” Int. J. Radiat. Oncol., Biol., Phys. 64, 625–634 (2006).
13. R. T. Droege “A practical method to routinely monitor resolution in digital images”. Medical Physics 10, 337-343 (1983)
14. R. Rajapakshe, K. Luchka. In “Quantitative Imaging in Oncology”(Eds: K. Faulkner, B. Carey, A. Crellin, R.M. Harrison). Proc. LH Gray Conf. on Quantitative Imaging in Oncology, Newcastle April 1995. BIR , London 1996 pp. 103-105.
15. Almond et al “AAPM’s TG-51 protocol for clinical reference dosimetry of high-energy photon and electron beams”. Medical Physics, 1847-1870(1999)
16. Osei, ErnstK et all “Dose assessment from an online kilovoltage imaging system in radiation therapy” J. Radiol. Prot. 29 (2009) 37–50
17. Bissonnette JP, Douglas J. Moseley, and David A. Jaffray “A quality assurance program for image quality of cone-beam CT guidance in radiation therapy” Medical Physics 35, 1807 (2008);
18. Yoo Sae et all. “A quality assurance program for the on-board imager” Medical Physics 33, 4431 (2006);
19. Garayoa J.,Castro P ” A study on image quality provided by a kilovoltage cone-beam computed tomography” Journal of Applied Clinical Medical Physics, Vol. 14 2013
20. Kim, Sangroh et all. “Estimation of Computed tomography dose index in cone beam computed tomography: mosefit measurements and monte carlo simulations” Health Physics May 2010, Volume 98, Number 5
21. Stock, Pasler,Georgeet all “Image quality and stability of image-guided radiotherapy (IGRT) devices: A comparative study” Radiotherapy and Oncology 93 (2009) 1–7
22. Srijit Kamath,1a William Song,2 Alexei Chvetsov,3 Shuichi Ozawa “ An Image Quality comparison study between XVI and OBI CBCT systems” Journal of Applied Clinical Medical Physics, Vol. 12 2011
23. Mohammad K. Islam, Thomas G. Purdie, Bernhard D. Norrlinger, Hamideh Alasti, Douglas J. Moseley, Michael B. Sharpe, Jeffrey H. Siewerdsen, and David A. Jaffray “Patient dose from kilovoltage cone beam computed tomography imaging in radiation therapy” Medical Physics 33, 1573 (2006);

.

**Figures**

Figure 1: The built in image guidance systems of the Novalis TX radiation delivery system are shown. (A) The kV source for the OBI, (B) The OBI a-Si flat panel detector, (C) The MV a-Si EPID mounted to an ExactArm.

Figure 2: The Varian OBI with the (A) QCkV-1 phantom and (B) RaySafe unsfors R/F detector in proper positioning for evaluation.

Figure 3: The EPID with the (A) QC-3 phantom and (B) PTW975 Semiflex Ion Chamber and SolidWater™ in proper positioning for evaluation.

Figure 4: The CATPHAN 504 CBCT evaluation phantom positioned for evaluation on the treatment couch. The CATPHAN is a cylindrical water equivalent phantom with outer diameter of 20 cm, inner diameter of 15 cm and 4 different inserted modules that can evaluate Uniformity, Noise, high contrast spatial Resolution, HU Constancy, Geometric Distortion and Slick thickness.

**Figures:**

**Figure 1**

**
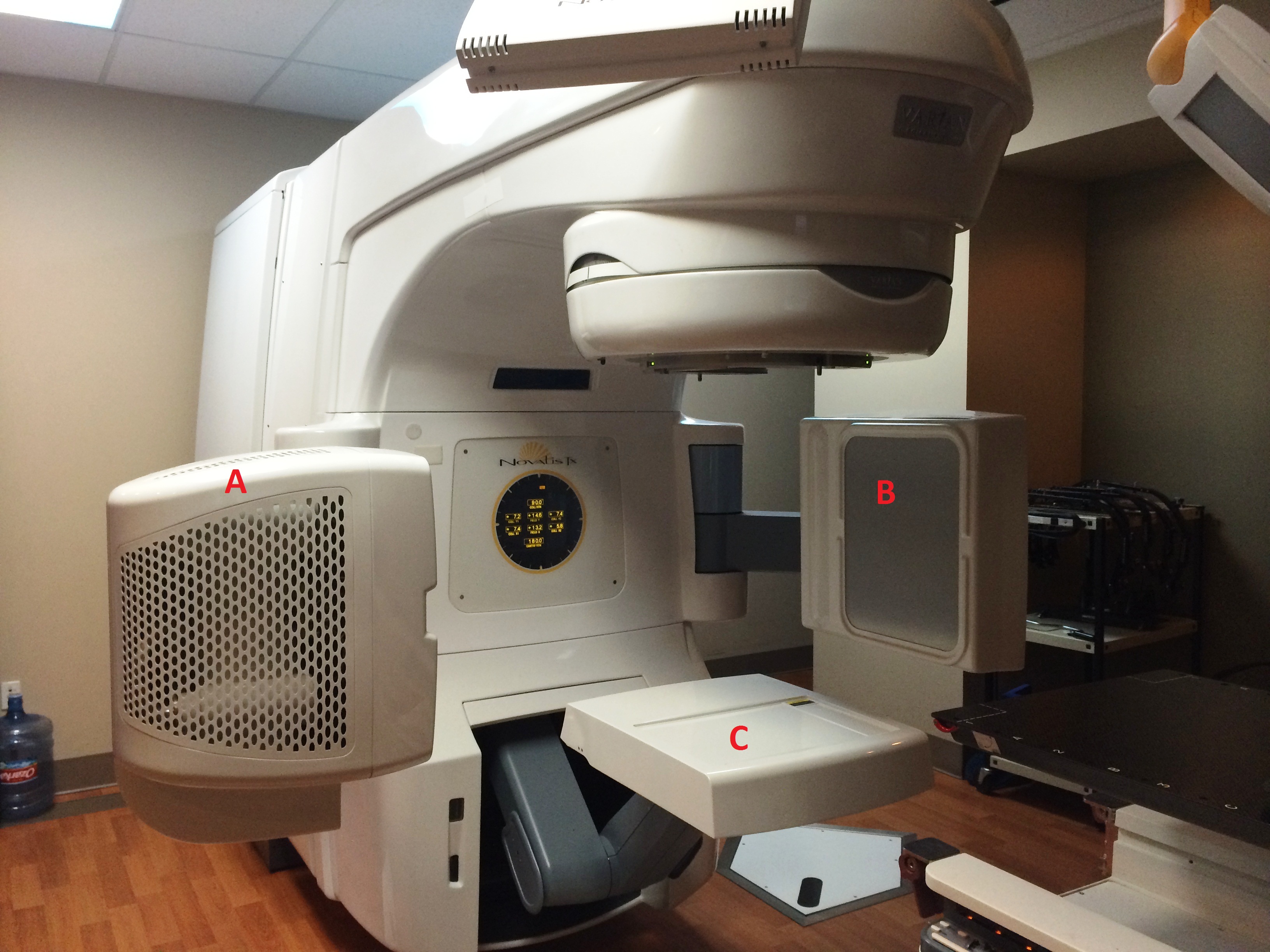
**

**Figure 2**

**
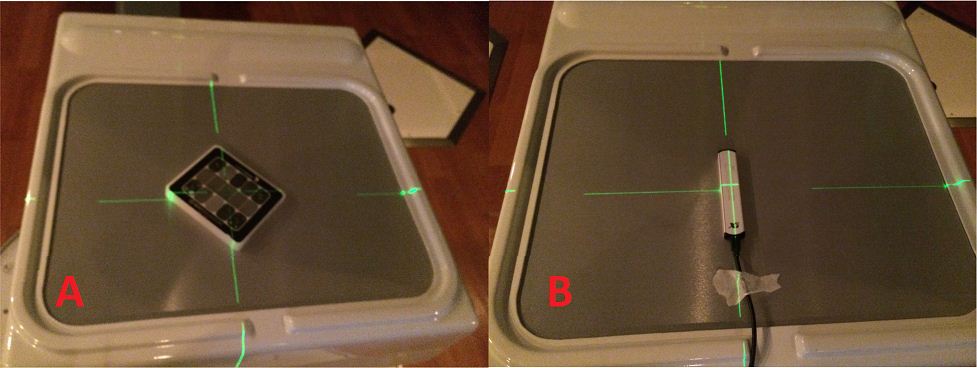
**

**Figure 3**

**
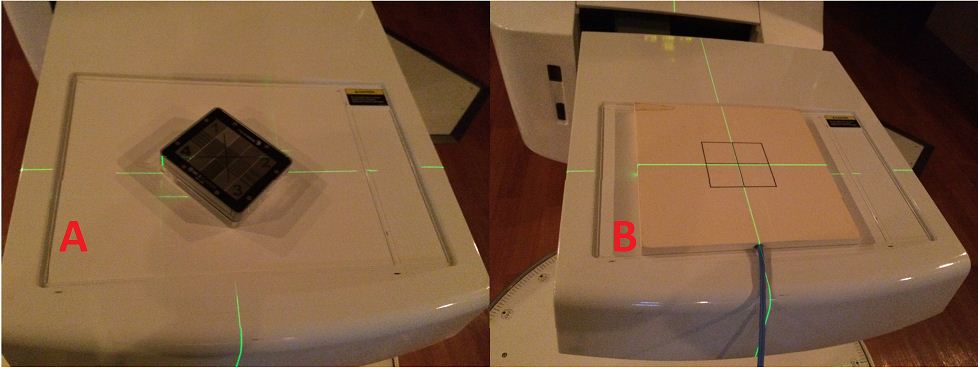
**

**Figure 4**

**
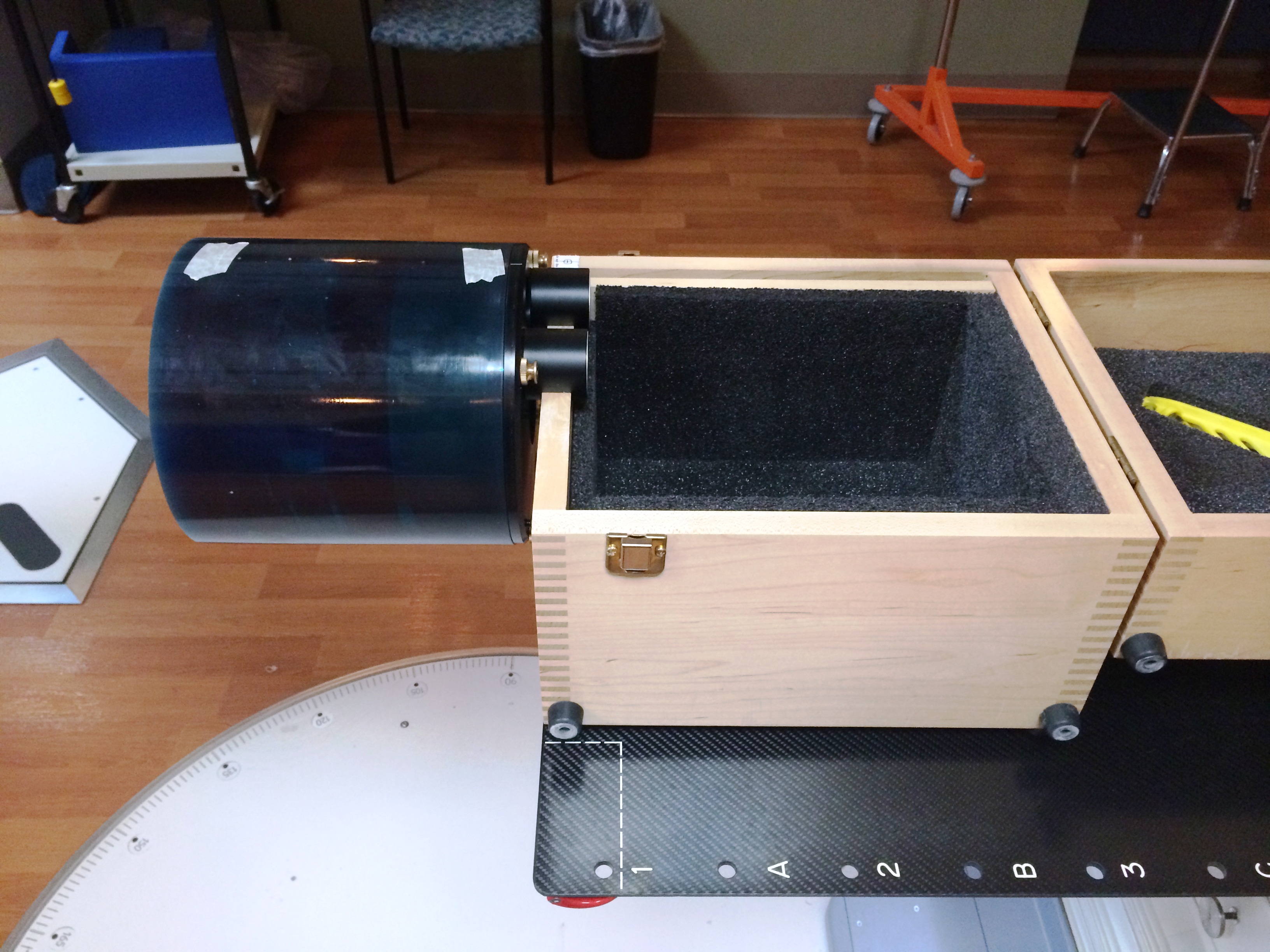
**

Tables:

| Table 1: Image Quality Scanning Parameters for the CBCT | | |
| --- | --- | --- |
|  | Half-Fan | Full-Fan |
| CBCT Mode | Pelvis | High quality Head |
| Patient orientation | Head first Supine | Head first Supine |
| Diameter PA and LR (FOV): | 26.0 cm | 24.0 cm |
| Reconstructed Volume | 512x512 | 512x512 |
| Slice Thickness: | 2.5 mm | 2.5 mm |
| Width (scan range): | 16 cm | 16 cm |
| kV: | 85 | 85 |
| mA: | 25 | 25 |
| ms: | 8 | 8 |

| Table 2: Normalized standard Deviations for all evaluated metrics | | | | | | | |
| --- | --- | --- | --- | --- | --- | --- | --- |
| **Planar Radiographic** | | | | | | | |
| **kV** | | | | **MV** | | | |
| f30(lp/mm) | 0.015 | kVp | 0.010 | f30(lp/mm) | 0.006 | Dose | 0.005 |
| f40(lp/mm) | 0.008 | Dose | 0.010 | f40(lp/mm) | 0.009 |  |  |
| f50(lp/mm) | 0.004 |  |  | f50(lp/mm) | 0.018 |  |  |
| **CBCT** | | | | | | | |
| **Full-Fan CBCT** | | | | | | | |
| **High contrast spatial resolution (lp/mm)** | | **HU constancy** | | **Geometric Distortion** | | **Dosimetric** | |
| f30 | 0.087 | Lung(PMP) | 0.023 | AP | 0.005 | **Dose** |  |
| f40 | 0.086 | Water(Poly) | 0.065 | LAT | 0.005 | Center | 0.004 |
| f50 | 0.074 | Bone(Derlin) | 0.020 | Slice thickness mean | 0.056 | Periphery | 0.004 |
| **Uniformity** | 0.061 | **Noise** | 0.063 |  |  |  |  |
| **Half-Fan CBCT** | | | | | | | |
| **High contrast spatial resolution(lp/mm)** | | **HU constancy** | | **Geometric Distortion** | | **Dosimetric** | |
| f30 | 0.110 | Lung(PMP) | 0.038 | AP | 0.006 | **Dose** |  |
| f40 | 0.116 | Water(Poly) | 0.058 | LAT | 0.005 | Center | 0.007 |
| f50 | 0.173 | Bone(Derlin) | 0.020 | Slice thickness mean | 0.059 | Periphery | 0.003 |
| **Uniformity** | 0.090 | **Noise** | 0.079 |  |  |  |  |

| Table 3:  Image Quality consistency Thresholds for the Planar Radiographic Modalities | | | | | |
| --- | --- | --- | --- | --- | --- |
| kV | | | **MV** | | |
|  | Warning | Action |  | Warning | Action |
| f30(lp/mm) | 2% | 4% | f30(lp/mm) | 2% | 4% |
| f40(lp/mm) | 1% | 3% | f40(lp/mm) | 1% | 3% |
| f50(lp/mm) | 1% | 3% | f50(lp/mm) | 1% | 3% |
| Dose | 1% | 2% | Dose | 1% | 2% |
| kVp | 1% | 2% |  |  |  |
| *Sample size of 60 measurements* | | | | | |

| Table 4:  Image Quality consistency Thresholds for the Full-Fan CBCT | | | |
| --- | --- | --- | --- |
|  | | **Warning** | **Action** |
| Uniformity | | 6% | 12% |
| Noise | | 6% | 12% |
| High contrast spatial resolution(lp/mm) | |  |  |
|  | f30 | 9% | 18% |
|  | f40 | 9% | 18% |
|  | f50 | 8% | 16% |
| HU Constancy | |  |  |
|  | Lung(PMP) | 3% | 6% |
|  | Water(Poly) | 6% | 12% |
|  | Bone(Derlin) | 2% | 4% |
| Geometric Distortion | |  |  |
|  | AP | 1% | 2% |
|  | LAT | 1% | 2% |
| Slice Thickness Mean | | 6% | 12% |
| Dose | |  |  |
|  | Center | 1% | 2% |
|  | Periphery | 1% | 2% |
| *Sample Size of 60 Measurements* | | | |

| Table 5:  Image Quality consistency Thresholds for the Half-Fan CBCT | | | |
| --- | --- | --- | --- |
|  | | **Warning** | **Action** |
| Uniformity | | 9% | 17% |
| Noise | | 8% | 16% |
| High contrast spatial resolution(lp/mm) | |  |  |
|  | f30 | 10% | 20% |
|  | f40 | 11% | 22% |
|  | f50 | 17% | 34% |
| HU Constancy | |  |  |
|  | Lung(PMP) | 4% | 8% |
|  | Water(Poly) | 6% | 12% |
|  | Bone(Derlin) | 2% | 4% |
| Geometric Distortion | |  |  |
|  | AP | 1% | 2% |
|  | LAT | 1% | 2% |
| Slice Thickness Mean | | 6% | 12% |
| Dose | |  |  |
|  | Center | 1% | 2% |
|  | Periphery | 1% | 2% |
| *Sample Size of 60 Measurements* | | | |

| Table 6:  Our Institutional Imaging QA Tolerances for Planar Radiographic imaging systems | | | | |
| --- | --- | --- | --- | --- |
| Frequency | **Quality Metric** | **Quality Check** | **Suggested Tolerance level**  **(Percent of Baseline)** | |
|  |  |  | Warning | Action |
| Monthly | High contrast spatial resolution (lp/mm) |  |  |  |
|  |  | f30 | >2% | >4% |
|  |  | f40 | >2% | >4% |
|  |  | f50 | >2% | >4% |
| Annual |  |  |  |  |
|  | Imaging quality | Imaging Dose | >1% | >2% |
|  |  | Imaging Energy(kV only) | >1% | >2% |

| Table 7:  Our Institutional Imaging QA Tolerances for CBCT imaging systems | | | | |
| --- | --- | --- | --- | --- |
| Frequency | **Quality Metric** | **Quality Check** | **Suggested Tolerance level**  **(Percent of Baseline)** | |
|  |  |  | Warning | Action |
| Monthly |  |  |  |  |
|  | Image quality | Geometrical Accuracy | >1% | >2% |
|  |  | High contrast spatial resolution (lp/mm) | >10% | >20% |
|  |  | Uniformity | >7% | >14% |
|  |  | Noise | >7% | >14% |
|  |  | CT number accuracy and stability | >6% | >12% |
| Annual |  |  |  |  |
|  | Imaging quality | Imaging Dose | >1% | >2% |
